# Associations of objectively measured physical activity and sleep in preschoolers aged 3 to 6 years

**DOI:** 10.3389/frsle.2024.1329774

**Published:** 2024-03-05

**Authors:** Laura Miller, Mya Dockrill, Penny V. Corkum, Sara F. L. Kirk, Michelle Stone

**Affiliations:** ^1^School of Health and Human Performance, Dalhousie University, Halifax, NS, Canada; ^2^Department of Psychology and Neuroscience, Dalhousie University, Halifax, NS, Canada; ^3^Department of Psychiatry, Dalhousie University, Halifax, NS, Canada; ^4^Healthy Populations Institute, Dalhousie University, Halifax, NS, Canada

**Keywords:** physical activity, sleep, objective measures, preschool-aged children, accelerometry, actigraphy

## Abstract

Research has demonstrated various negative effects of poor sleep on overall health in children. Engaging in physical activity during the day is often recommended to help children sleep better. Limited research has examined this recommendation for preschool children, although physical activity is generally supported as a healthy sleep practice. When measuring physical activity and sleep, objective measures (e.g., accelerometers) are recommended as opposed to subjective measures (e.g., parental reports). The purpose of the current study was to examine the relationship between objectively measured sleep (key variables included sleep efficiency, sleep onset latency, and sleep duration) and physical activity (operationalized as mean daily total physical activity) among preschool-aged children in Nova Scotia, Canada. Children (*n* = 29) wore a wrist accelerometer to objectively measure sleep and wore an accelerometer on their waist to measure physical activity for nine consecutive days. Overall, linear regression analyses demonstrate physical activity as a predictor of sleep efficiency but not total sleep time or sleep onset latency among preschool-aged children. Future research should examine the causal relationships between sleep efficiency and physical activity by conducting interventions to increase physical activity and determining the impact on sleep efficiency.

## 1 Introduction

Approximately 25% of children experience some type of behavioral sleep problem (Owens, 2007), including difficulties with falling asleep, staying asleep, or waking up too early (5th ed.; DSM-5-TR; American Psychiatric Association, 2022). Insufficient sleep is considered to be a public health epidemic that is often unrecognized and under-reported (Chattu et al., 2019). Sleep problems in children are associated with a wide range of physical and mental health deficits including increased fatigue and daytime sleepiness (Sadeh, 2007), poor immune system functioning (United States Department of Health Human Services, 2020), and deficits in cognitive and emotional functioning (Sadeh et al., 2003; Vriend et al., 2015). To help prevent these adverse physical and psychosocial health outcomes, it is important to better understand how to improve sleep among children, as the odds of sleep problems worsening into adulthood are significantly higher among poor sleepers in childhood and adolescence (Fernandez-Mendoza et al., 2022). Sleep problems tend to persist from preschool to childhood and into adulthood (Byars et al., 2012; Mamun et al., 2012; Reidy et al., 2016), and therefore intervening during the preschool-aged years is critical to prevent unhealthy sleep practices from becoming established (Craigie et al., 2011).

There is growing recognition of the important relationship between sleep and physical activity (PA). The relationship between PA and sleep is evident in the Canadian 24-h Movement Guidelines, which recognizes the inherent associations between PA, sedentary behavior (SB), and sleep, and their contributions to health outcomes (Tremblay et al., 2017). These guidelines conceptualize movement on a continuum from sleep to high levels of PA and were developed through multiple systematic reviews examining relationships within and among the movement behaviors and their health indicators (Carson et al., 2017a; Chaput et al., 2017; Kuzik et al., 2017). As an individual's movement behaviors saturate the entire 24-h period, a change in any movement behavior is likely done at the expense of another (Tremblay et al., 2017). Recent research suggests that approximately one-fourth to one-third of Canadian children and youth sleep less than recommended for optimal health and wellbeing (Chaput and Janssen, 2016; Michaud and Chaput, 2016). The 2020 ParticipACTION Report Card identified factors associated with insufficient sleep in youth including sociodemographic factors, excessive screen use, lack of parental monitoring, or social activities (ParticipACTION, 2020). These findings highlight the importance of understanding more about the interrelationships between children's movement behaviors.

PA is widely supported as a healthy sleep practice and is often recommended to improve sleep in children (Fonesca et al., 2021). Research suggests there could be a beneficial relationship between PA and sleep among children, however, this research is limited, particularly related to preschool-aged children. Research has demonstrated an effect of physical activity on sleep quality of children (St. Laurent et al., 2020; Zhao et al., 2023). There are two current reviews examining the association between PA and sleep in children, with one including seven studies examining this association in preschool-aged children under 5 years old (Janssen et al., 2020), and the other including five studies with a mean age in the preschool years (three to 6 years) (Antczak et al., 2020). Janssen et al. (2020) found an overall significant association between PA and most sleep outcomes in preschoolers, whereas Antczak et al. (2020) found little association between PA and sleep. These inconsistencies in results make it challenging to draw firm conclusions regarding the relationship between PA and sleep. One confounding factor that is likely contributing to these inconsistencies is how researchers are measuring sleep and PA.

When measuring PA and sleep among children, objective measures (e.g., accelerometers) are recommended as opposed to subjective measures (e.g., parental reports) (Trost et al., 2005; Fitzhugh, 2015). Specifically, accelerometers worn on the waist to measure PA (Trost et al., 2005) and on the wrist to measure sleep (Slater et al., 2015) are advised. Waist accelerometers are recommended to measure PA, as these accelerometers are better able to measure energy expenditure during walking and running compared to wrist accelerometers (Trost et al., 2005), whereas wrist location is thought to be optimal for measuring sleep (Martin and Hakim, 2011). Additionally, when measuring PA and sleep objectively, it is recommended that these movement behaviors be collected for a minimum of 4 to 7 days to ensure reliable estimates of PA and sleep variables (Hinkley et al., 2012; Taylor et al., 2015). Previous research has not always followed these recommendations.

Previous research has used both objective and subjective tools, and data have not always been collected for the recommended time period (Williams et al., 2014; Plancoulaine et al., 2015; Tatsumi et al., 2015). In a review and meta-analysis, Antczak et al. (2020) identified 47 studies examining the relationship between PA and sleep in children aged 3 to 13. Seven of the studies had samples in the preschool age range, and of these, 2 studies followed recommended length of data collection between four and seven days and only 1 study used objective measures of both PA and sleep. The one study that used both objective measures of PA and sleep found a positive relationship between PA counts per minute and percent sleep (the percentage of total sleep between sleep onset and sleep end time). Another study, not included in Antczak et al. (2020) review, also found an association between objective measures of PA and sleep, with higher PA levels associated with poorer sleep habits (Eythorsdottir et al., 2020). As waist accelerometers are recommended for measuring PA, and wrist accelerometers are recommended for measuring sleep, future research should incorporate the recommended tools for measuring PA and sleep respectively (Trost et al., 2005; Slater et al., 2015). Further research, which follows these measurement guidelines is needed to elucidate the relationship between PA and sleep in preschool-aged children. Therefore, the purpose of the current study was to examine the relationship between PA and sleep in a sample of preschool-aged children living in Nova Scotia (NS), Canada, using objective measures of PA and sleep. Based on the existing literature, it was hypothesized that PA would be a predictor of sleep efficiency, sleep duration, and sleep onset latency among preschool-aged children.

## 2 Materials and methods

### 2.1 Participants and study design

This within-subjects research is part of a larger study, the Physical Literacy in the Early Years (PLEY) project, examining the efficacy of an outdoor loose parts intervention to promote active outdoor play among preschool-aged children (Houser et al., 2019). All data used in the current study were collected before the PLEY project intervention. The current study examined the relationship between objectively measured total PA (% of day spent active) and objectively measured total sleep time (TST), sleep onset latency (SOL), and sleep efficiency (SE) (see *Measures* for operational definitions of sleep variables).

Participants included children (*n* = 34) between the ages of 3 and 6 years and their parents in Nova Scotia (NS), Canada. As sleep behavior can vary with medical and psychiatric conditions (Freeman et al., 2020; Fadzil, 2021), children with known medical or psychiatric conditions were excluded from this research. Screening for exclusion criteria took place during the consent process by asking parents about the existence of any medical or psychiatric conditions. Participants in the PLEY project were recruited from regulated childcare centers in NS, and participants for the current study were recruited from the PLEY project sample. Thus, all participants in the current study were in care environments during the day. Data collection took place between October 2017 and March 2018. The study was approved by the Dalhousie University Research Ethics Board (#2016-3924).

### 2.2 Procedure

A parent survey was distributed to parents via email. This survey was part of the larger PLEY project (Houser et al., 2019), and contained questions related to the participant's age, sex, socio-economic status (SES), PA, sedentary time, physical literacy, and sleep. The sleep questions (TST, SOL, and SE) used to describe the sample were from the Child Sleep Habits Questionnaire (Owens et al., 2000) (see Appendix A). Parents were asked to complete the sleep questions based on their children's sleep during a typical week.

The main objective measures for this study included an accelerometer to measure daytime activity and an actigraph to measure sleep. Children wore an Actigraph GT3X+ accelerometer on their waist to objectively measure PA for nine consecutive days. Accelerometers were put on in the morning after getting out of bed and were removed at bedtime. We included all days of the week (i.e., weekday and weekend) for reliable estimates of PA (Hinkley et al., 2012), although research has suggested that reliable estimates of PA can be obtained in young children with or without the inclusion of a weekend day (Bisson et al., 2019). Parents were also provided with an accelerometer wear-time log to report times each day the accelerometer was not worn. Children's sleep was measured objectively using a MicroMini-Motionlogger actigraph. The actigraph was worn on the non-dominant wrist during the same nine-day measurement period as the waist accelerometer. In order to capture the full sleep period, children placed the actigraph on their wrist 1 h before bedtime and removed it 1 h after their final wake-up time in the morning. Parents filled out a Sleep Log to set bedtime time, lights out time, and waketimes each morning and night, which was used to aid in the scoring of the actigraph data (Corkum, 1996) (see Appendix B).

To be included in the analysis, each child required a minimum of 4 days with 6 hours of PA accelerometer wear time each day; a weekend day was not necessary for inclusion (Hinkley et al., 2012). Children required a minimum of 4 days of sleep data gathered via the actigraph (Taylor et al., 2015). Data from individual days were removed from the analysis if the day did not have valid data for both PA and sleep.

### 2.3 Measures

#### 2.3.1 Demographic survey

The PLEY project incorporated an author-made parent survey to collect demographic data. In the current study, this information was used to describe the children with respect to their age, sex, and to describe their family demographics, including socioeconomic status (SES; as measured by family income), ethnicity, and family structure (i.e., number of parents in the home, number of people in the home).

#### 2.3.2 Sleep and activity questionnaire

The Sleep and Activity Questionnaire was administered as part of the larger PLEY study and collected information about the parent and child's physical activities as well as the child's sleep. The questions asking about sleep were modeled after some of the questions from the Child Sleep Habits Questionnaire (CSHQ; Owens et al., 2000). The sleep questions included were those that asked the broadest questions about the child's sleep duration, sleep onset, sleep quality, waking, and daytime alertness. This information was collected to describe the sample based on parent report. The data was not used as outcome data.

#### 2.3.3 Actigraph GT3X+ accelerometer

Children's time spent sedentary, and time spent in light and moderate-vigorous physical activity were measured objectively using ActiGraph GT3X+ accelerometers (ActiGraph LLC, Pensacola, FL), worn on an elastic waistband around the child's hip. To improve compliance and ensure data quality, parents were given an instruction sheet that explained how to attach the accelerometer over their child's right hip and when to remove the device (night-time sleep, bathing/swimming). Parents were also given an accelerometer wear time log (Appendix C) to report times the accelerometer was removed from the child throughout each day over the nine-day measurement period. Parents also reported the time their child put the accelerometer on in the morning and what time the accelerometer was removed before bedtime each day.

Accelerometer-measured PA data were reduced and analyzed using ActiLife (Version 6; ActiGraph LLC). Accelerometer data reduction decisions were consistent with a previous study of Canadian preschoolers (Carson et al., 2017b) and equivalent to previously published PLEY project data (Stone et al., 2019). Data were collected in 15s epochs. Non-wear time was defined as ≥20 min of consecutive zeros (Carson et al., 2017b). Sedentary time was defined as ≤ 100 counts/min, light PA as 100–1,679 counts/min and moderate to vigorous PA as ≥1,680 counts/min (Janssen et al., 2013). Accelerometer data were then classified into minutes per day and % of day spent sedentary, in light PA (LPA), in moderate to vigorous PA (MVPA), and in PA of any intensity (total PA), for each valid day. Specifically, accelerometer data for all valid days were then summed and divided by the number of valid days to create an individual average daily score for each PA variable (represented in minutes/day). To calculate the % of day spent sedentary, in light PA, and in MVPA, average minutes per day spent sedentary, in LPA, and in MVPA, were each divided by the average daily wear time (in minutes). Total PA was calculated by summing the average daily minutes of LPA and MVPA; and presented as the % of the day spent active by dividing by the average daily wear time (in minutes). The conversion of minutes per day spent sedentary, in LPA and MVPA, and total PA, to the respective % of the day spent in these various PA intensities, was done to account for the differences in accelerometer wear time between children and made these PA variables (%'s) more comparable across participants. As the 24-h movement guidelines suggest children accumulate at least 180 min of PA of any intensity (i.e., LPA of MVPA) per day (Tremblay et al., 2017), total PA was used as the predictor variable to align with this guideline.

#### 2.3.4 MicroMini-Motionlogger

Children's sleep was measured objectively using a MicroMini-Motionlogger actigraph (Ambulatory Monitoring Inc; https://www.ambulatory-monitoring.com/motionlogger-actigraphs). This actigraph is a type of accelerometer that has a wristwatch appearance and was worn on the non-dominant wrist as per guidelines (Sadeh et al., 1994). The actigraph uses an accelerometer to measure motor activity (data are collected in 1-min epochs), and this movement is used to determine if the child is awake or asleep. The Sadeh algorithm (Sadeh et al., 1994) uses an 11-min window, including the five previous and five future epochs to detect sleep. Detected movements are translated into digital counts and are entered into the sleep index formula. If the result of the formula is >-4, the epoch is considered asleep (Sadeh et al., 1994). The data were then extracted using the Act Millennium operational software, and summary analysis was computed using the Action-W2 which uses a validated sleep estimation algorithm (Sadeh et al., 1989). Summary analysis included the variables of interest for this study: TST, SOL, and SE. TST is defined as the amount of time spent sleeping between sleep onset and final wake-up time. This variable excludes the amount of time spent awake after sleep onset. SOL is defined as the amount of time it takes to fall asleep after “lights out.” Finally, SE is defined as the percentage of time spent sleeping between “down for the night” (the time when the child was in bed with the intention to fall asleep) and “up for the day” (the wake-up time).

#### 2.3.5 Sleep and activity logs

A sleep log (Appendix B), based on a previous sleep diary used in previous research (Corkum, 1996), was used to help score the actigraph sleep data. The sleep log, completed by parents, contained questions pertaining to various sleep variables such as bedtime, lights out time, sleep onset, night awakenings, and final wake-up time. Information regarding any times the actigraph was removed each day throughout the 9-day period was also gathered via this log. The times of day reported for lights out and wake-up time were used when scoring the “down” interval (i.e., the time in bed ready for sleep interval) on the actigraph.

An activity log (Appendix C) was used by parents to report times that the accelerometer was removed from the child throughout each day over the 9-day measurement period. Parents also reported the time their child put the accelerometer on in the morning and what time the accelerometer was removed before bedtime each day.

### 2.4 Statistical analysis

Descriptive statistics were used to present demographics, PA data, and sleep data. All data from the objective measures of sleep and PA were checked for linearity and normality before running the data analyses. A series of three linear regression analyses were used to determine the relationship between total PA (% of day spent active) and TST, SOL, and SE. One analysis was run for each sleep variable (as the outcome variable) and PA was the predictor variable. Age and sex were used as covariates and as such were put into the model first to determine if they were significant. Sleep outcome assessors were blind to participants' PA exposure. Statistical significance was defined as *p* < 0.05. In terms of missing sleep data, there was an average of 27.22% of days missing across participants (*M* = 2.45, *Range:* 0–5 days missing. In terms of missing PA data, only 1 participant had 1 day of missing data. There was an average of 0.33% of days missing across participants (*M* = 0.03, *Range:* 0–1 days missing).

## 3 Results

### 3.1 Sample descriptives

Data were collected from 34 children, although 5 children were removed from the analysis due to errors in data collection or insufficient days of data. The final sample for analysis included 29 children. One child's data was removed from the demographic survey due to missing data. Based on data from the demographic survey, children were on average 4.29 years old (SD = ± 0.81 years; range = 3 to 6 years) and there were 20 males and 9 females. Overall, the sample mostly had a high income, high parental education level, was mostly of European descent, had two parents, and mostly there were 4 individuals living in the home (see Table 1 for demographic data). The parent-reported sleep information indicated that 60.7% of children obtained 10–13 h of sleep during weekday nights, 57.1% of children obtained 10–13 h of sleep during weekend nights, with 53.6% falling asleep within 20 min, and 21.4% waking up more than once during the night. The majority of the sample met PA guidelines of 180 min per day (82.1%) and were sedentary <4 h a day (78.6%). Descriptive data for parent report of their child's sleep and PA can be found in Table 2. The sample's sleep and PA data based on objective measures were consistent with parent reports in that the sample was generally comprised of children who slept well and engaged in age-appropriate levels of PA. Based on the Actigraph GT3X+ accelerometer data, children spent a daily average of 188.3 min being sedentary, 277.0 min in LPA, 222.3 min in MVPA, and 494.4 min in total PA representing 72.8% of the day engaged in PA. On average, the children slept for 504.4 min per day, took 26.9 min to fall asleep, and had a sleep efficiency of 85.8%. Additional data (standard deviations and range) and accelerometer wear time can be found in Table 3.

**Table 1:** Demographic data (*n* = 28).

	**Frequency (%)**
Household income	
<$20,000	0 (0)
$21,000–$40,000	0 (0)
$41,000–$60,000	1 (3.6)
$61,000–$80,000	3 (10.7)
$81,000–$100,000	0 (0)
>$100,000	19 (67.9)
Unsure	1 (3.6)
Prefer not to answer	4 (14.2)
Level of parental education	
Completed Junior/Middle School	0 (0)
Completed Secondary/High School	1 (3.6)
Completed Community/Technical College	4 (14.2)
Completed Undergraduate University Degree	6 (21.5)
Completed Graduate/Advanced University Degree	17 (60.7)
Prefer not to answer	0 (0)
Parental ethnicity	
Aboriginal	1 (3.6)
European descent	19 (67.9)
East Asian descent	1 (3.6)
Not listed	3 (10.7)
Multiple	4 (14.2)
Family structure	
Couple with child/children	27 (96.4)
Single parent family with child/children	1 (3.6)
Number of people living in house	
3	9 (32.1)
4	12 (42.8)
5	6 (21.5)
6	1 (3.6)

**Table 2:** Parent-reported measures of children's sleep (child sleep habits questionnaire), sedentary behavior (parent survey), and physical activity (parent survey) (*n* = 28).

	**Frequency (%)**
Weekday night sleep duration	
<8 h	0 (0)
8 to <10 h	11 (39.3)
10 to 13 h	17 (60.7)
>13 h	0 (0)
Weekend night sleep duration	
<7 h	0 (0)
7 to <10 h	12 (42.9)
10 to 13 h	16 (57.1)
>13 h	0 (0)
Does your child fall asleep within 20 min?	
Rarely (0 to 1/week)	3 (10.7)
Sometimes (2 to 4/week)	10 (35.7)
Usually (5 to 7/week)	15 (53.6)
Does your child awaken more than once during the night?	
Rarely (0 to 1/week)	22 (78.6)
Sometimes (2 to 4/week)	6 (21.4)
Usually (5 to 7/week)	0 (0)
Unknown	0 (0)
Child sitting time	
No sitting	4 (14.3)
<1 h	1 (3.6)
1 to 2 h	13 (46.4)
3 to 4 h	4 (14.3)
>4 h	6 (21.4)
Does your child meet the PA guidelines of 180 min per day?	
Yes	23 (82.1)
No	5 (17.9)

**Table 3:** Accelerometry-measured sedentary behavior, physical activity, and sleep characteristics (*n* = 29).

**Characteristics**	**(SD; Range), *n* = 29**
PA variables (average)	
SB, min	188.3 (55.8; 50.9–300.1)
LPA, min	277.0 (48.7; 167.3–432.1)
MVPA, min	222.3 (47.8; 127.2–364.4)
Total PA, min	494.4 (88.0; 290.5–796.5)
Accelerometer wear time, min	679.9 (117.5; 362.1–1095.6)
Average PA and SB per day, % of day	
SB, %	27.2 (6.9; 11.7–45.4)
LPA, %	40.0 (4.0; 33.7–47.7)
MVPA, %	32.8 (6.3; 19.2–51.8)
Total PA, %	72.8 (6.9; 54.6–88.3)
Sleep variables (average)	
TST, min	504.4 (58.0; 397.3–628.9)
SOL, min	26.9 (17.2; 5.6–70.0)
SE, %	85.8 (6.8; 69.3–98.2)

Values are presented as means (ranges). PA, physical activity; SB, sedentary behavior; LPA, light physical activity; MVPA, moderate to vigorous physical activity; Total PA, total physical activity; TST, total sleep time; SOL, sleep onset latency; SE, sleep efficiency.

**Figure 1:**
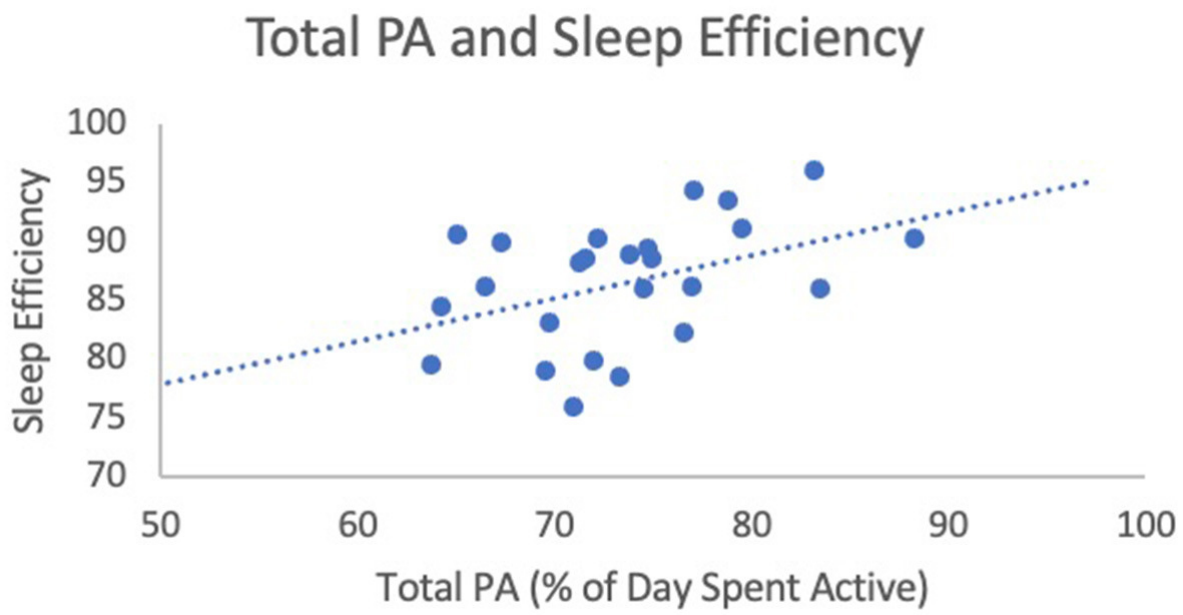
Total PA (% of day spent active) and sleep efficiency. This figure illustrates the relationship between total PA (predictor variable) and sleep efficiency (outcome variables).

**Figure 2:**
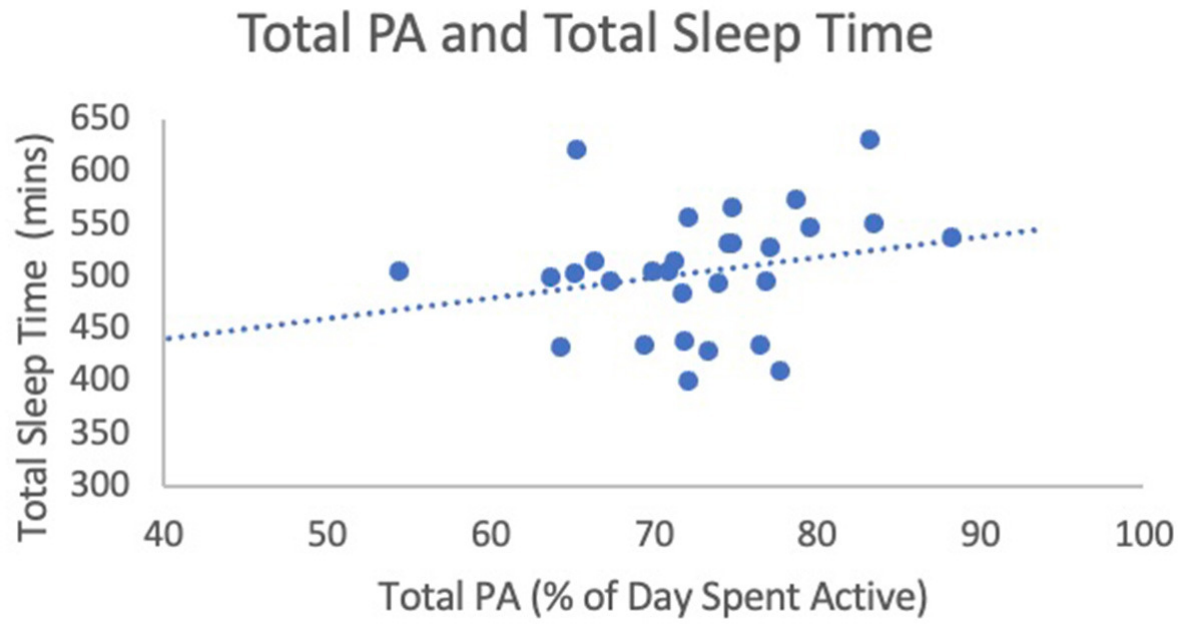
Total PA (% of day spent active) and sleep efficiency. This figure illustrates the relationship between total PA (predictor variable) and sleep efficiency (outcome variables).

**Figure 3:**
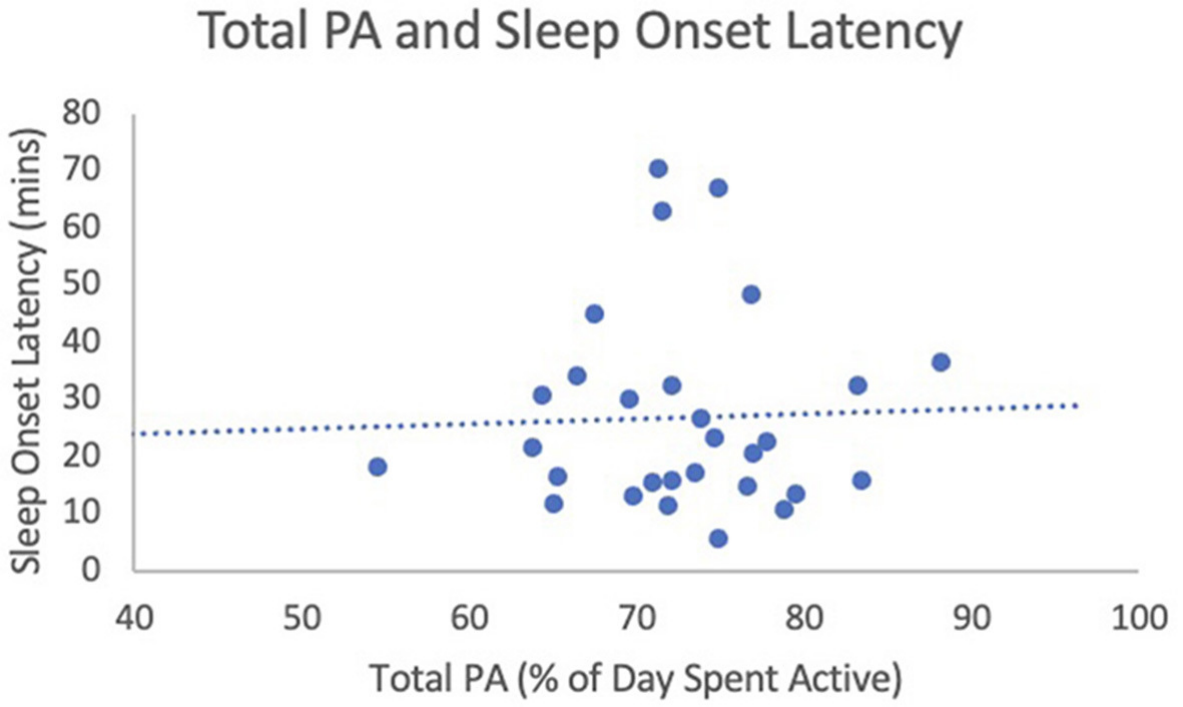
Total PA (% of day spent active) and sleep efficiency. This figure illustrates the relationship between total PA (predictor variable) and sleep efficiency (outcome variables).

When analyzing the relationship between total PA (% of day spent active) and SE, results of a linear regression analysis found that total PA was a significant predictor of SE [F_(1, 25)_ = 5.07, *b*_1_ = 0.37, *p* = 0.03, 95% CI (0.03, 0.70)]. Age and sex did not add to the model and these variables were therefore removed. For each percent increase in total PA, SE increased by 0.37% (Figure 1). The effect size was equal to 0.61, indicating a strong effect. Linear regression revealed no relationship between total PA and TST [F_(1, 28)_ = 1.55, *b*_1_ = 1.96, *p* = 0.22, 95% CI (−1.27, 5.19); see Figure 2]. Age and sex did not add to the model, so these variables were removed. The effect size was equal to 0.24, indicating a small effect. Linear regression revealed that total PA was not a significant predictor of SOL [F_(1, 29)_ = 0.03, *b*_1_ = 0.09, *p* = 0.86, 95% CI (−0.90, 1.07); see Figure 3]. Age and sex did not add to the model and these variables were therefore removed. The effect size was equal to 0.05, indicating a small effect.

## 4 Discussion

The purpose of this research was to better understand the relationship between PA and sleep among preschool-aged children, following best practice guidelines for measuring both PA and sleep. The current study examined the relationship between total PA and TST, SOL, and SE. PA was measured objectively via accelerometry for nine consecutive days. Sleep was measured objectively via actigraphy during the same 9-day measurement period as the PA data collection.

It is important to note, in this study, all children obtained more than the recommended PA guidelines (Tremblay et al., 2017). This contrasts with data from the 2022 ParticipACTION Report Card reporting that only 28% of children and youth are meeting the physical activity recommendations with the Canadian 24-Hour Movement Guidelines for children and youth (ParticipACTION, 2022). Additionally, children participating in this research were on average within the “may be appropriate” sleep duration range of 8 to 14 h of sleep per night (National Sleep Foundation, 2004). Moreover, children's sleep variables (SE, TST, SOL) were also generally within normal ranges (Tétreault et al., 2019; Kocevska et al., 2021). This is in contrast to recent findings that a substantial number of children and youth (around 25%) are not meeting their sleep recommendations (Roberts et al., 2017).

Results revealed that total PA was a significant predictor of objectively measured sleep SE, but not TST or SOL. There is limited research examining the relationship between PA and objectively measured sleep, and results of previous studies are inconsistent; it is therefore difficult to draw comparisons between these findings and previous research. These are consistent with results from two previous studies examining sleep subjectively (i.e., parental report) among preschool-aged children (Hense et al., 2011; Plancoulaine et al., 2015). Four previous studies used waist-worn accelerometers to measure both PA and sleep in preschool-aged children; one study found no association between MVPA and TST (Duraccio and Jensen, 2017), while another study found an inverse relationship between total PA and TST (Williams et al., 2014). Other studies have found favorable associations between MVPA and sleep (Tatsumi et al., 2015; Taylor et al., 2015). A previous study (Nixon et al., 2009) examined the relationship between PA and SOL among 7-year-old children; results revealed an inverse relationship between total PA and SOL, inconsistent with the current study's findings.

Potential factors including screen time, mealtimes, and time spent in daycare during the day may have influenced the association between sleep and PA. Further, the association between PA and SE may have been observed due to a number of possible mechanisms. PA can help to decrease arousal, anxiety, and depressive symptoms, thereby helping individuals to sleep (particularly those that experience sleep difficulties) (Passos et al., 2011). Further, PA can affect the circadian rhythm which is endogenously generated but is modulated by external cues such as sunlight. For individuals that have difficulty sleeping, PA can shift the circadian rhythm's timing, thereby helping individuals to sleep (Guilleminault et al., 1995). PA's effect of shifting the circadian rhythm is especially noticeable when PA occurs outdoors as sunlight influences melatonin levels.

### 4.1 Strengths

By using an objective measure to examine both PA and sleep, this research is of methodological soundness compared to many previous studies examining PA and sleep among children (Iwasaki et al., 2010; Fitzhugh, 2015). Furthermore, to our knowledge, this study was one of the first of its kind, as it used the recommended tools for measuring PA and sleep (i.e., waist accelerometer and wrist accelerometers, respectively) for the expected duration of 4 to 9 days (Trost et al., 2005). Objectively measured PA and sleep data were collected for nine consecutive days, and children required a minimum of 4 days of sufficient data to be included in analyses. This time period of data collection meets recommendations for this methodology (Hinkley et al., 2012; Taylor et al., 2015). Additionally, to be included in data analysis, children required a minimum of 6 hours of accelerometer wear time each day (Hinkley et al., 2012).

### 4.2 Limitations

This study is limited by its sample size, as it included only 29 children. Other studies identified in the Janssen et al. (2020) systematic review that examined total PA and sleep ranged from 48 to 826 children under 5 years old (Janssen et al., 2020). The other review ranged from 48 to 8,542 preschool-aged children with a mean age between 3 and 6 years (Antczak et al., 2020). Had the sample size been larger, total PA may have been a significant predictor of other sleep variables. For example, although the relationship between total PA and TST was non-significant, TST increased as children spent a greater proportion of their day active (see Figure 1). The trend was therefore in the direction that was hypothesized. It could be that had the sample size been larger, this trend may have been significant. Also, given the small sample size, we had to select one predictor variable for PA and selected what seemed to be the most robust predictor based on the literature. A larger sample size would have allowed us to include multiple PA predictors. Moreover, a larger sample would allow for novel statistical analyses such as crossed-lagged analyses that could examine the inter-relationship between sleep and PA on a day-to-day basis and also provide more insight into the causal relationships between PA and sleep. A recent study using this methodology found SE impacted PA the next day, but PA did not impact SE (St. Laurent et al., 2022). While objective measures were used in this study, both sleep and activity were measured using wrist location rather than having activity measured by the recommended waist location. Further, the current study examined the percentage of worn accelerometer time as there was a wide range of wear time across participants (362 min to 1,095 min) and therefore percentages may be skewed.

Families participating in the study were of higher SES (higher income and parental education), and primarily of European descent, therefore results of the study may not necessarily be generalizable to larger populations of families of varying SES and ethnicities. Also, the current study only examined the relationship between PA and sleep, whereas other research has examined other activity variables beyond PA variables (e.g., sedentary behavior) and sleep among preschool children (Duraccio and Jensen, 2017). Further, we did not account for naptimes in this study, which may have impacted the results as children may have napped at the childcare centers and/or at home. We also were not able to ensure all children had weekend and weekday data, which may have also skewed the data given the differences between weekday and weekend sleep and PA.

Lastly, our result suggests that, in our sample, sleep was quite good overall and so was physical activity, which given the high rates of sleep problems and low physical activity in the general populations may indicate a biased sample (Hale et al., 2009; van Rossem et al., 2012; Jarrin et al., 2014).

### 4.3 Future directions

It is recommended that additional research is conducted using a similar protocol (i.e., measuring sleep and PA using the gold-standard assessment methods), but with a larger sample size. This would allow for more variables to be examined (e.g., to examine other PA variables as predictors of sleep), as well as more novel analyses to be conducted (e.g., crossed-lagged analyses), which could provide insights into the causal relationships between PA and sleep. Conducting similar research in a more diverse sample with children who are not meeting recommended sleep and daily activity levels would be interesting given that our findings were in a sample mostly comprised of affluent Caucasian families whose children were sleeping and being physically active for the recommended daily times. The findings may be even more salient in a more diverse sample of children who were not meeting recommended sleep and activity levels. Given that a recent meta-analysis (Zhao et al., 2023) found evidence that PA improves sleep quality in young children, it would be important to evaluate the effects of interventions on these variables and the directionality of effects. If the relationship between PA and sleep is supported in future research, examining the effectiveness of interventions on these variables would be of great interest.

## 5 Conclusion

It is known that most children in today's society are not meeting sleep recommendations and are suffering from associated health consequences (ParticipACTION, 2022). Understanding how to improve sleep through healthy sleep practices (e.g., PA) that promote optimal development and improved overall health is essential. This study is significant as it adds to the limited knowledge base about PA and sleep using objective measures following best practice guidelines among preschool-aged children and demonstrates a significant relationship between PA and sleep quality even in a homogeneous not at-risk sample. Future experimental research is needed to draw firm conclusions regarding the relationship between PA and sleep among preschool-aged children.

## Data availability statement

The raw data supporting the conclusions of this article will be made available by the authors, without undue reservation.

## Ethics statement

The studies involving humans were approved by Dalhousie University Research Ethics Board (ethics approval #: 2016-3924). The studies were conducted in accordance with the local legislation and institutional requirements. The participants provided their written informed consent to participate in this study.

## Author contributions

LM: Conceptualization, Data curation, Methodology, Formal analysis, Project administration, Writing – original draft, Writing – review & editing. MD: Writing – review & editing, Formal analysis. PC: Conceptualization, Methodology, Resources, Supervision, Writing – review & editing. SK: Conceptualization, Methodology, Supervision, Funding acquisition, Writing – review & editing. MS: Conceptualization, Methodology, Resources, Supervision, Funding acquisition, Writing – review & editing.
