# Supplementary material for: Associations of objectively measured physical activity and sleep in preschoolers aged 3 to 6 years
Source: Front Sleep. 2024 Mar 5;3:1329774. doi: 10.3389/frsle.2024.1329774 (PMC12713936; doi:10.3389/frsle.2024.1329774)
Supplement: Supplementary file 1 [file Table_1.DOCX]

Supplementary Material

Associations of objectively measured physical activity and sleep in preschoolers aged 3 to 6 years

Laura Miller, Mya Dockrill, Penny Corkum, Sara Kirk, Michelle Stone*

*** Correspondence:** Michelle Stone: michelle.stone@dal.ca

# Supplementary Data

## Supplementary Appendices

**Appendix A**

Questions used from the Child Sleep Habits Questionnaire (Owens et al., 2000)

1) On a weekday, how much sleep does your child usually get?

a) Less than 8 hours, b) less than 10 hours, c) 10 to 13 hours, or d) more than 13 hours

2) Does your child typically fall asleep within 20 minutes after going to bed?

a) Rarely – 0 to 1/week, b) Sometimes – 2 to 4/week, or c) Usually – 5 to 7/week

3) Does your child awake more than once during the night?

a) Rarely – 0 to 1/week, b) Sometimes – 2 to 4/week, c) Usually – 5 to 7/week, or d) Unknown

**Appendix B**

Sleep Log

Please answer the following questions to the best of your ability for each day.

DAY 1-9:

- Today’s date (mm/dd/yy) ___________________________
- What time did your child put on the actigraph? _______
- What time did your child get into bed? _______
- What time was “lights out”? _______
- What time did your child fall asleep? _______
- What time did your child wake up the following the morning? _______

*Note.* Parents completed the same questions each day.

**Appendix C**

Accelerometer Activity Log
